# Supplementary material for: An interpretable hybrid predictive model of COVID-19 cases using autoregressive model and LSTM
Source: Sci Rep. 2023 Apr 25;13:6708. doi: 10.1038/s41598-023-33685-z (PMC10126574; doi:10.1038/s41598-023-33685-z)
Supplement: Supplementary file 1 — Supplementary Information. [file 41598_2023_33685_MOESM1_ESM.pdf]

Supplementary material

Structure of neural network. We detail the structure of LSTM regression network and the cell gate below.

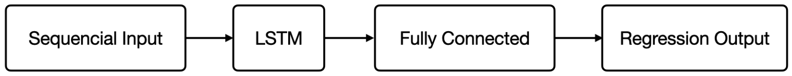

Supplementary figure 1. This diagram illustrates the architecture of a simple LSTM network for regression. The network starts with a sequence input layer followed by an LSTM layer. The network ends with a fully connected layer and a regression output layer.

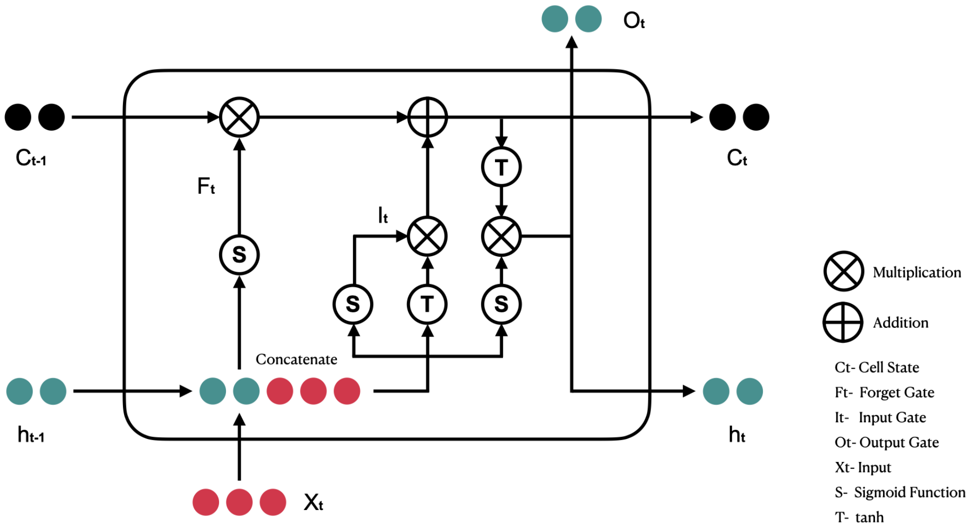

Supplementary figure 2. Structure of a LSTM cell that describes the cell state c_t (green circles, dim =2) and hidden state h_t (red circles, dim =2) and various gates[1].

California data

Missing data

We study the number of daily COVID-19 cases in 8 California counties: Los Angeles, San Diego, San Francisco, Santa Barbara, Fresno, Sacramento, Ventura, and Riverside, from 2020-02-01 to 2022-09-05.
These data can be found on the official website of California State Government[2]. Specifically, we collect data from the statewide COVID-19 cases deaths tests. Since there is only one missing value, collected in Los Angeles, we drop the observations of the same date from all counties' data. The final dataset contains consecutive time series data, collected from the 8 California counties, each with 948 observations collected in the past three years. We will use Y_t to denote the number of COVID-19 cases at time t, where t is an integer index that takes value in $\{1,2,\ldots,948\}$.

Statistical analysis

Although we primarily focus on the number of daily COVID-19 cases, we have also analyzed other variables in the dataset, such as the number of reported cases, as illustrated in Supplementary figure 3. By examining the correlations between these variables and the number of daily cases, we aim to provide insights for future research.

As depicted in the plot, there is a positive correlation between daily COVID-19 cases and timely data, such as daily total tests and reported cases. However, cumulative data appears to play a less significant role in predicting daily cases, with

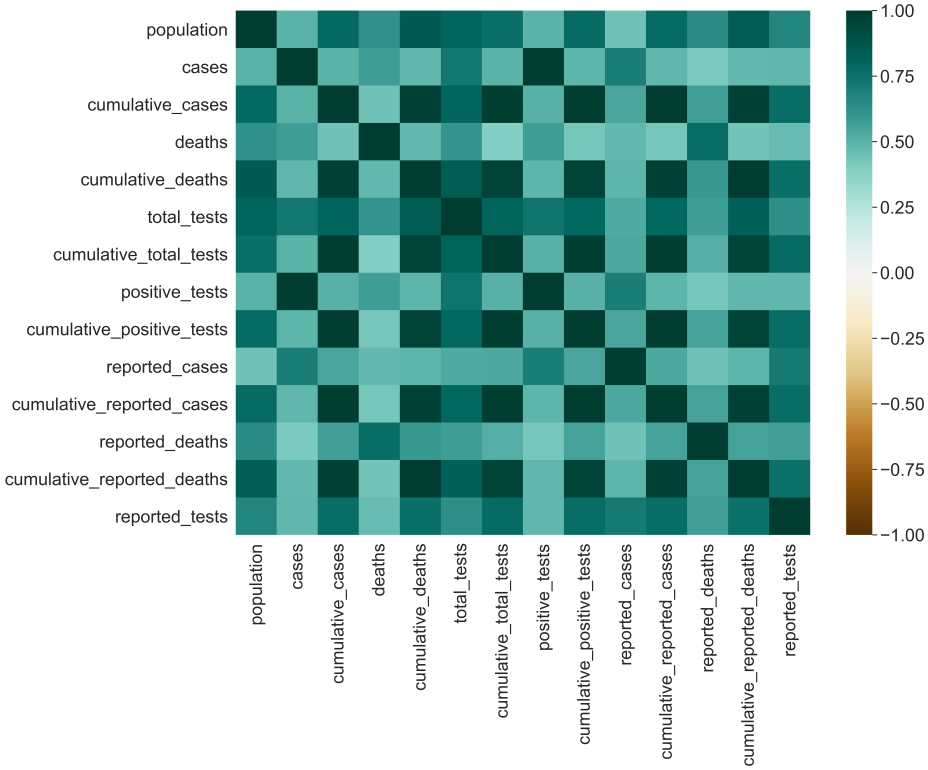

Supplementary figure 3. Examining the correlation between each variable and the number of cases. The variables with darker colors are more strongly correlated with the number of cases.

correlation values below 0.5. Among the variables that are positively related to daily cases, daily total tests have the highest correlation coefficient, even more than reported cases. On the other hand, daily deaths have the lowest positive correlation coefficient.

Training

In this section, we provide details on how we fit the models discussed in section Methods, and introduce the experiment setups for performance evaluation.

Data preprocessing and preparation

Smoothing. Recall that we denote Y_t as the number of COVID-19 cases at time t. We adopt the standard smoothing techniques below to smooth out the irregular roughness between time steps:

$$\bar{Y}_t := \frac{1}{\tau}\left(\sum_{i=t-\tau+1}^{t} Y_i\right),$$

where τ is the smoothing lag number. In our study, we choose $\tau = 7$ by exploiting the day of week effect: the number of observations each date is influenced by which day of a week it is. For example, the observations of COVID-19 case detection are empirically higher on Fridays, which might be attributed to the fact that people have time to do the testing on Fridays. The day of week effect has long been observed in healthcare and the stock market. To avoid the day of week effect, we smoothed the data by using the average of observations from 6 previous dates and the current date. We will use the smoothed data as the ground truth throughout the study.

Experiment setup for a single trial

In reality, we often aim at making reliable and timely predictions based on an appropriate length of data. Furthermore, it is well-known that the different variants of COVID-19 could result in different transmission mechanisms, leading the joint distribution of confirmed cases to shift from time to time. Therefore, it is not advised to train the model using all historical data since pandemic. Therefore, we cut the time series into different pieces and split each piece into training set and test set. We then

apply the model training and testing on each piece, which we call a trial. For each trial, we do a three-step data preprocessing:

Differencing: Statistical methods such as AR have guaranteed performance on stationary time series. However, the raw data are typically not stationary. Differencing can help us stabilize the mean of a time series by removing changes in the level of a time series, and therefore eliminating (or reducing) trend and seasonality. This can make the differenced time series stationary. Specifically, we keep differencing data until stationarity is achieved by standard tests such as Augmented Dickey Fuller Unit Root Test (ADF)[3] or Kwiatkowski Phillips Schmidt Shin Test (KPSS)[4]. We found a consistent stationarity among different trials with only one differencing operation.
Rescale. After differencing the data, we rescale the training data into the $[-1,1]$ with:

$$Y_{\text{train,scaled}} = \frac{Y_{\text{train}} - \mu_{\text{train}}}{Y_{\text{max,train}} - Y_{\text{min,train}}}$$

where μ_{train} denotes the mean of Y_{train}, $Y_{\text{max,train}}$ denotes the maximum of Y_{train}, $Y_{\text{min,train}}$ denotes the minimum of Y_{train}, and $Y_{\text{train,scaled}}$ denotes training data after scaling. We also apply the same re-scaling map to the testing data:

$$Y_{\text{test,scaled}} = \frac{Y_{\text{test}} - \mu_{\text{train}}}{Y_{\text{max,train}} - Y_{\text{min,train}}}$$

Thus the testing data does not affect the selection of our scalar. We train the model on $Y_{\text{train,scaled}}$ and make predictions with $Y_{\text{test,scaled}}$. Finally, we apply the inverse rescaling map to retrieve the original scale, and thus make comparison with the original data.

Reshaping. After differencing and rescaling the data, we transform the data into supervised learning form as shown below in the Supplementary table 1.

	Input	Output
Row 1	$[Y_{t-n}, \cdots, Y_{t-n+1}]$	Y_{t-n+p}
Row 2	$[Y_{t-n+1}, \cdots, Y_{t-n+2}]$	$Y_{t-n+p+1}$
Row 3	$[Y_{t-n+2}, \cdots, Y_{t-n+3}]$	$Y_{t-n+p+2}$
$\vdots$	$\vdots$	$\vdots$

Supplementary table 1. The input-output data format. Here p is the lag number.

This step is also conducted on the testing data for all three models and the training data for the LSTM model and the hybrid model.

We are now ready to conduct the experiment on a single trial. To begin with, each trial has 88 continuous observations. We apply a first order differencing to make the trial data stationary, at the cost of losing one observation. Now we have 87 observations. The first 62 would be used as the training data: notice that our training size is 63, since the 62 differenced values are derived from 63 observations. The remaining 25 values will be used to make a testing data matrix.

We apply the rescaling on the training and test data. Then we reshape the testing data of length 25 to a matrix of size $(18,8)$. For each of the 18 rows, the first 7 values are model inputs, which would return a single predicted value to us: an prediction for the rescaled $Y_t^{(1)}$, where the superscript 1 refers to the first order differencing. Let us denote this prediction with:

$$\widehat{Y}_{t,\text{scaled}}^{(1)} \tag{1}$$

We derive an prediction of the ground truth Y_t from (1). First, we scale (1) back to the original scale by applying the inverse function of rescaling to it. Then (1) becomes an prediction of $Y_t^{(1)}$, say $\hat{Y}_t^{(1)}$. We now retrieve an estimation of the ground truth Y_t with:

$$\hat{Y}_t = \widehat{Y}_t^{(1)} + Y_{t-1} \tag{2}$$

Notice that all observations before Y_t are known. Since we obtain one estimation from each row, we will end up with a list of 18 estimated values. We assess the model by comparing these 18 estimations with the 18 corresponding ground truth values.

Choice of hyper-parameters

In this section, we detail how we choose the hyper-parameters for each predictive model.

AR: We used lag number 7 for the sake of interpretability, since 7 is the number of days in a week. There could exist more scientific methods to select the lag number. For example, we may check the Bayesian information criterion (BIC). BIC is a class of information criteria to measure the goodness of fit of a statistical model. It builds on the concept of entropy and can weigh the complexity of the estimated model against the goodness of fit of this model to the data. This information helps to assess the model's parameters and how well the model performed.

LSTM: We use batch number 1, epoch number 100, and units 1. The neural network is trained on mean square error, with optimizer adam. The fully connected layer is activated by the default linear function. Since the performance is well enough for our purpose, we do not tune the model further.

Hybrid model: This neural network is the addition of 2 layers: an AR layer and a LSTM layer, after being weighted by a trainable coefficient between 0 and 1. We use the same set of hyperparameters as we do in the AR model and the LSTM model. As a result, we have some flexibility to tune to make the performance better.

Additional evaluation measures

RMSE

RMSE measures the difference between the forecast and the ground truth. It is a suitable metric because it removes the influence of data size and yields a result that has the same units as the input data: in other words, it is interpretable and explicit, and we can use it to compare our models to others' work, done on different datasets. RMSE is calculated by

$$\mathrm{RMSE} = [\sum_{i=1}^{n} (\widehat{Y}_t - Y_{\mathrm{true}})^2 / n]^{\frac{1}{2}} \tag{3}$$

MAE

MAE is another metrics commonly used to evaluate regression models. It measures the mean absolute difference between the forecasts and the ground truth values. MAE is calculated by

$$\mathrm{MAE} = \frac{1}{n} \sum_{i=1}^{n} |\widehat{Y}_t - Y_{\mathrm{true}}| \tag{4}$$

Just as MAPE, for both RMSE and MAE, a method with lower value is preferred.

Additional prediction results

Beside MAPE, we evaluate and compare RMSE and MAE values of the models on the same dataset as in section Results. As shown in Supplementary table 2 and 3, the hybrid model has the best general performance, indicated by its lowest RMSE and MAE for each county, and it is usually the best model on the last trial. Besides, the hybrid model usually has the lowest standard error, suggesting its performance is stabler than that of its competitors.

References

1. Karim, R. Animated rnn, lstm and gru (2020).
2. CHHS Open Data. Statewide covid-19 cases deaths tests (2022).
3. Mushtaq, R. Testing time series data for stationarity. *SSRN* (2011).
4. Shin, Y. & Schmidt, P. The kpss stationarity test as a unit root test. *Econ. Lett.* **38**, 387–392, DOI: https://doi.org/10.1016/0165-1765(92)90023-R (1992).

	County	AR	LSTM	LSTM (Double)	hybrid
General performance	Los Angeles	265.490 (115.718)	233.677 (91.393)	260.866 (91.526)	**203.208** (92.471)
	San Diego	68.809 (27.350)	60.559 (24.826)	67.053 (24.769)	**51.445** (20.784)
	San Francisco	11.880 (4.441)	10.107 (3.518)	11.561 (3.823)	**7.943** (2.520)
	Santa Barbara	8.926 (2.853)	7.314 (2.257)	8.585 (2.359)	**6.732** (2.181)
	Fresno	18.918 (4.284)	16.649 (4.311)	17.964 (4.441)	**13.635** (2.988)
	Sacramento	24.864 (9.058)	21.880 (8.272)	22.844 (7.632)	**18.183** (6.809)
	Ventura	18.159 (5.825)	16.219 (6.295)	17.918 (6.137)	**13.557** (4.644)
	Riverside	49.038 (15.131)	41.535 (13.401)	49.035 (14.847)	**35.246** (11.030)
Latest performance	Los Angeles	93.059	76.594 (1.578)	76.909 (2.473)	**74.740** (0.506)
	San Diego	26.829	24.277 (0.277)	23.987 (0.392)	**23.312** (0.088)
	San Francisco	6.393	5.498 (0.057)	5.458 (0.043)	**5.439** (0.011)
	Santa Barbara	4.719	4.443 (0.031)	**4.377** (0.051)	4.391 (0.012)
	Fresno	8.687	7.765 (0.090)	8.369 (0.374)	**7.544** (0.017)
	Sacramento	14.179	11.957 (0.094)	**11.765** (0.133)	12.269 (0.054)
	Ventura	7.404	6.274 (0.038)	6.243 (0.065)	**6.126** (0.025)
	Riverside	23.653	18.311 (0.354)	18.228 (0.557)	**18.217** (0.210)

Supplementary table 2. RMSE for each model on each county. General performance is averaged on all trials. The inconsistent performances of neural networks have been compensated by the small step value, which is 7. The Latest performance is on the latest trial, from 2022-06-10 to 2022-09-05. The results for LSTM, LSTM double and hybrid are each averaged on 100 runs, to compensate the inconsistent performances of neural networks. The value in parenthesis is the standard error. AR has 0 or small standard error for the same trial. The hybrid model is usually the best in performance and has the lowest standard error.

	County	AR	LSTM	LSTM (Double)	hybrid
General performance	Los Angeles	208.591 (91.929)	190.269 (76.486)	220.444 (79.963)	**154.930** (72.476)
	San Diego	55.252 (22.012)	47.836 (19.699)	55.140 (20.268)	**40.233** (16.836)
	San Francisco	9.149 (3.235)	8.166 (2.865)	9.769 (3.326)	**6.074** (1.860)
	Santa Barbara	7.069 (2.369)	5.674 (1.806)	7.097 (2.022)	**5.204** (1.737)
	Fresno	15.026 (3.425)	13.537 (3.654)	14.733 (3.751)	**10.646** (2.342)
	Sacramento	19.814 (7.339)	17.362 (6.833)	18.547 (6.431)	**13.934** (5.259)
	Ventura	14.653 (4.692)	12.919 (5.100)	15.080 (5.349)	**10.804** (3.893)
	Riverside	39.736 (12.249)	34.055 (11.550)	40.854 (12.716)	**27.577** (8.649)
Latest performance	Los Angeles	59.253	55.311 (2.053)	52.760 (2.986)	**48.597** (0.527)
	San Diego	16.291	15.600 (0.293)	16.101 (0.769)	**14.848** (0.085)
	San Francisco	5.119	4.045 (0.085)	4.054 (0.066)	**3.918** (0.016)
	Santa Barbara	3.346	2.902 (0.025)	**2.847** (0.043)	2.897 (0.007)
	Fresno	5.423	5.012 (0.130)	5.876 (0.446)	**4.721** (0.014)
	Sacramento	9.026	7.276 (0.112)	**7.118** (0.148)	7.371 (0.051)
	Ventura	5.029	4.147 (0.040)	4.112 (0.063)	**3.918** (0.026)
	Riverside	18.046	13.123 (0.473)	12.287 (0.647)	**11.456** (0.159)

Supplementary table 3. MAE for each model on each county. General performance is averaged on all trials. The inconsistent performances of neural networks have been compensated by the small step value, which is 7. The Latest performance is on the latest trial, from 2022-06-10 to 2022-09-05. The results for LSTM, LSTM double and hybrid are each averaged on 100 runs, to compensate the inconsistent performances of neural networks. The value in parenthesis is the standard error. AR has 0 or small standard error for the same trial. The hybrid model is usually the best in performance and has the lowest standard error.
